# Understanding the null hypothesis (H_0_) in non-inferiority trials

**DOI:** 10.1186/s13054-017-1685-2

**Published:** 2017-05-06

**Authors:** Jihad Mallat

**Affiliations:** 1 Department of Anesthesiology and Critical Care Medicine, Centre Hospitalier du Dr. Schaffner de Lens, Lens cedex, France; 2 Intensive Care Unit, Centre Hospitalier d’Arras, Arras, France; 3 Centre Hospitalier du Dr. Schaffner, Service de Réanimation polyvalente, 99 route de La Bassée, 62307 Lens cedex, France

I read with great interest the article by Zhou et al. [1] aiming to test whether a lactate-decreasing resuscitation protocol (lactate strategy), compared with central venous oxygen saturation-oriented resuscitation therapy (ScvO_2_ strategy), would decrease mortality among septic shock patients.

It is not clear why the authors performed a non-inferiority trial (NIT) whereas the primary objective of the study was to establish whether the lactate strategy was “superior” to the ScvO_2_ strategy [1]. Even though evidence of superiority can be claimed from NITs, there are several fundamental differences between superiority trials and NITs [2]. Whereas superiority trials aim to determine whether a new intervention is superior to the best available one, NITs seek to demonstrate that the new intervention is no worse than the comparator by more than a pre-specified, small amount. This amount is known as the non-inferiority margin, or delta (Δ). The null hypothesis (H_0_) of superiority trials asserts that there is no true difference between the interventions, and the alternative hypothesis (H_1_) states that there is a difference between the interventions. A type I error is the error of rejecting H_0_ when it is actually true. A type II error is a failure to reject H_0_ when in fact H_1_ is true. NITs, by contrast, have a H_0_ that the new intervention is inferior or worse than the old by more than − Δ (it is inferior). The H_1_ to be proven is that the new intervention is inferior to the standard intervention by less than − Δ (it is not inferior; Fig. 1) [2]. Thus, the definitions of type I and type II errors are reversed for NIT.

**Fig. 1:**
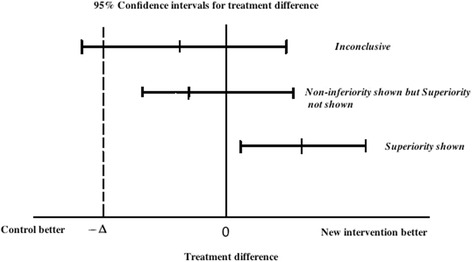
Different possible scenarios of the results of a non-inferiority clinical trial. ∆ is the non-inferiority margin

In this study, the authors claimed the superiority of the lactate strategy over the ScvO_2_ strategy because the lactate group had a significantly lower mortality compared with the ScvO_2_ group (18.3 versus 27.9%, *P* = 0.033). However, the *P* value that is calculated in NITs is special and is called the *P* value for non-inferiority, which differs from the *P* value for superiority [3]. The finding that *P* value of the difference in mortality was 0.033 means only that H_1_ is accepted and the lactate strategy is not inferior to the ScvO_2_ strategy. To be able to claim superiority, the 95% confidence interval of the mortality difference, which is not provided in this study, should exclude zero (Fig. 1).

Moreover, the non-inferiority margin in this study was 15% [1]. However, the authors did not provide any justification as to why they chose 15 rather than 10% as used in a previous trial [4].

## Abbreviations

NIT: Non-inferiority trial
ScvO_2_: Central venous oxygen saturation
